# Combined mutations of NKX2-1 and surfactant protein C genes for refractory low oxyhemoglobin saturation and interstitial pneumonia: A case report

**DOI:** 10.1097/MD.0000000000019650

**Published:** 2020-03-20

**Authors:** Rui Gu, Guangyong Ye, Yimin Zhou, Zhou Jiang

**Affiliations:** a Sir Run Run Shaw Hospital; b Women's Hospital, School of Medicine, Zhejiang University, Hangzhou, Zhejiang, China.

**Keywords:** interstitial pneumonia, low oxyhemoglobin saturation, NKX2-1 gene, surfactant protein C gene

## Abstract

**Rationale:**

Mutations of the NKX2-1 gene are associated with brain-lung-thyroid syndrome, which is characterized by benign hereditary chorea, hypothyroidism, and pulmonary disease with variable presentation. Surfactant protein C (SFTPC) gene mutations result in chronic interstitial lung disease in adults or severe neonatal respiratory distress syndrome.

**Patient concerns:**

Recurrent hypoxemia was observed shortly after birth in a baby at a gestational age of 40 weeks and birth weight of 3150 g. The need for respiratory support gradually increased. He had hypothyroidism and experienced feeding difficulties and irritability.

**Diagnosis:**

Genetic examination of the peripheral blood revealed combined mutations of the NKX2-1 and SFTPC genes.

**Interventions:**

The patient was administered respiratory support, antibiotics, low-dose dexamethasone, supplementary thyroxine, venous nutrition, and other supportive measures.

**Outcomes:**

The patient's guardian stopped treatment 3 months after commencement of treatment, due to the seriousness of his condition and the patient died.

**Lessons:**

Combined mutations of NKX2-1 and SFTPC genes are very rare. Thus, idiopathic interstitial pneumonia with hypothyroidism and neurological disorders require special attention.

## 1 Introduction

Brain-lung-thyroid syndrome is cause by mutation of the NKX2-1 gene, which causes the destruction of major organs, that is, the lungs, thyroid glands, and nervous system. The mutation is closely associated with different types of clinical manifestations. A recent review showed that 50% of cases involved the brain, lung, and thyroid, the brain and thyroid were involved in 30% of cases, while only neuropathy was observed in 13% and 7% did not exhibit any neurological symptoms.^[1]^ A novel heterozygous insertion (c.915_916insC) in exon 3 leads to severe respiratory distress and congenital hypothyroidism in the neonatal period and subsequent hypotonia and ataxia.^[2]^ Monti et al^[3]^ reported a novel heterozygous 29-base pair deletion (c.278_308del) in exon 2 with mild clinical presentation including movement disorders, choreoathetosis, and hypothyroidism. Williamson et al^[4]^ reported benign hereditary chorea with hypothyroidism caused by a novel missense mutation (c.626G > C; p.Arg209Pro) in NKX2-1. Kleinlein et al^[5]^ revealed a heterozygous 29-base pair deletion (c.278_306del29) in a term male infant with respiratory failure requiring mechanical ventilatory support, and mild hypothyroidism.

Surfactant protein C gene (SFTPC) mutations result invariable clinical manifestations, including chronic interstitial lung disease (ILD) in adults, or severe respiratory distress syndrome in neonates.^[6]^ The mutation appears as a single de novo mutation or may be inherited from the parents, who may be symptomatic or asymptomatic. The surfactant protein C (SP-C) regulated by the SFTPC gene is concerned with pulmonary surfactant homeostasis, normal pulmonary structure and function, and most importantly, gas exchange.^[7]^

We identified a novel mutation of NKX2-1 combined with SFTPC mutation in a term male infant with ILD, persistent low oxyhemoglobin saturation, congenital hypothyroidism, feeding difficulty, irritability, and convulsion.

## 2 Case presentation

The patient was born to non-consanguineous healthy parents at a gestational age of 40 weeks with premature rupture of the fetal membranes by 46 hours, first degree turbidity of amniotic fluid, normal Apgar scores, and a birth weight of 3150 g. After 5 minutes of birth, the infant was admitted to the neonatal intensive care unit due to cyanosis. The neonate exhibited dyspnea, skin cyanosis, and cried loudly but fever, nausea, vomiting, or convulsion were absent.

The infant was kept warm, provided oxygen with ahead box, provided with nutrition by the venous route. He developed pneumonia and pneumothorax suddenly, which were treated with ampicillin and cefotaxime. The patient experienced severe dyspnea with high oxygen requirement 9 days after birth. The pneumothorax improved but the pneumonia exacerbated. Cefotaxime was replaced by meropenem for better anti-infective effect. Nasal continuous positive airway pressure (NCPAP) was used to aid breathing. The pneumonia changed to the bilateral diffuse infiltration type after 10 days of antibiotic treatment. The oxyhemoglobin saturation was unstable (between 25% and 30%) with supplemental oxygen and NCPAP was required. The patient was administered low-dose dexamethasone for 9 days. The patient's respiratory status improved and NCPAP was replaced by nasal catheter oxygen inhalation. The infant needed nasal catheter oxygen inhalation needed again, 29 days after birth and the oxyhemoglobin saturation decreased significantly when the patient cried. Neurological symptoms including feeding difficulty and irritability emerged, which did not improve during hospitalization. Oxyhemoglobin saturation fluctuated, despite the use of nasal catheter oxygen inhalation. Respiratory distress and perioral cyanosis were observed when the infant cried, and thus, NCPAP was administered again. The patient developed interstitial pneumonia, which was treated by azithromycin administration, once daily. The parents decided to leave our hospital and went to Zhejiang Children's Hospital for further treatment, 31 days after birth. The patient experienced a convulsion with no fever or diarrhea on reaching that hospital. After 2 months’ hospitalization, the baby developed recurrent cyanosis with a declining heart rate. The oxyhemoglobin saturation decreased to 50%, and external cardiac compression was performed immediately. A high frequency ventilator was needed continuously to facilitate breathing, but the parents decided to stop treatment.

## 3 Methods

This report has been approved by the ethics committee of the Sir Run Run Shao Hospital of Zhejiang University. The patient's guardian provided informed consent for publication of this case.

### 3.1 Laboratory examination

Thyroid function tests were performed after 20 days of birth, which revealed elevated thyroid stimulating hormone (TSH) levels >100 mU/L (normal 0.35–4.94) and low thyroxine levels (T3 0.67 ng/mL and T4 4.67 μg/dL; normal range, 0.7–1.48 ng/mL and 4.87–11.72 μg/dL, respectively). Levothryoxine (Euthyrox) treatment was initiated and the serum TSH normalized after 8 days of treatment. After 31 days of birth, anti-cytomegalovirus IgM and pathogen DNA were positive, sputum culture was positive for *Escherichia coli*, and blood culture demonstrated fungal infections. Blood gas analysis revealed respiratory acidosis. Routine blood indices, liver and kidney function, electrolytes, myocardial enzyme levels were within their respective normal ranges. Serum antibodies were negative for Toxoplasma species and the rubella virus.

### 3.2 Imaging studies

**Figure 1:**
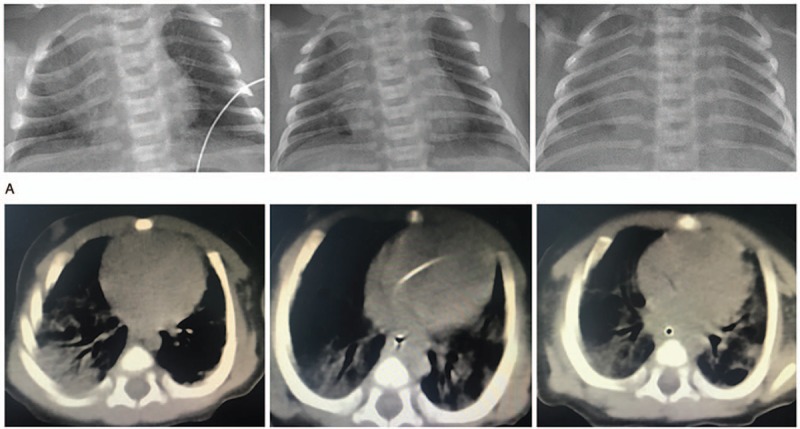
Chest radiographs and CT scan of the patient. (A) Chest radiographs taken at days 9, 16, 28 of life demonstrating a substantial improvement in pneumothorax and continuous deterioration to bilateral diffuse interstitial pneumonia. (B) CT scan taken on day 32 of life. CT = computed tomography.

Initially, chest radiography showed pneumothorax and pneumonia on the right side. Chest radiographs showed diffuse ground glass opacity, which increased from days 9 through 28 of life (Fig. 1A). The severity of pneumonia increased, while pneumothorax improved 9 days after birth. Chest radiography revealed diffuse infiltration in both lungs after 7 days of antibiotic treatment. It revealed mild reduction in radiopacity of both lungs after 12 days. Significantly reduction in radiopacity was observed with both lungs after 19 days, which confirmed the presence of interstitial pneumonia. Echocardiography revealed an ostium second umatrial septal defect (Ф0.5CM) and patent ductus arteriosus (Ф0.17CM). Computed tomography (CT) of the brain did not reveal any abnormality. CT (Fig. 1B) of the lung obtained after 1 month demonstrated the possibility of bronchopulmonary dysplasia.

### 3.3 Karyotype analysis

**Figure 2:**
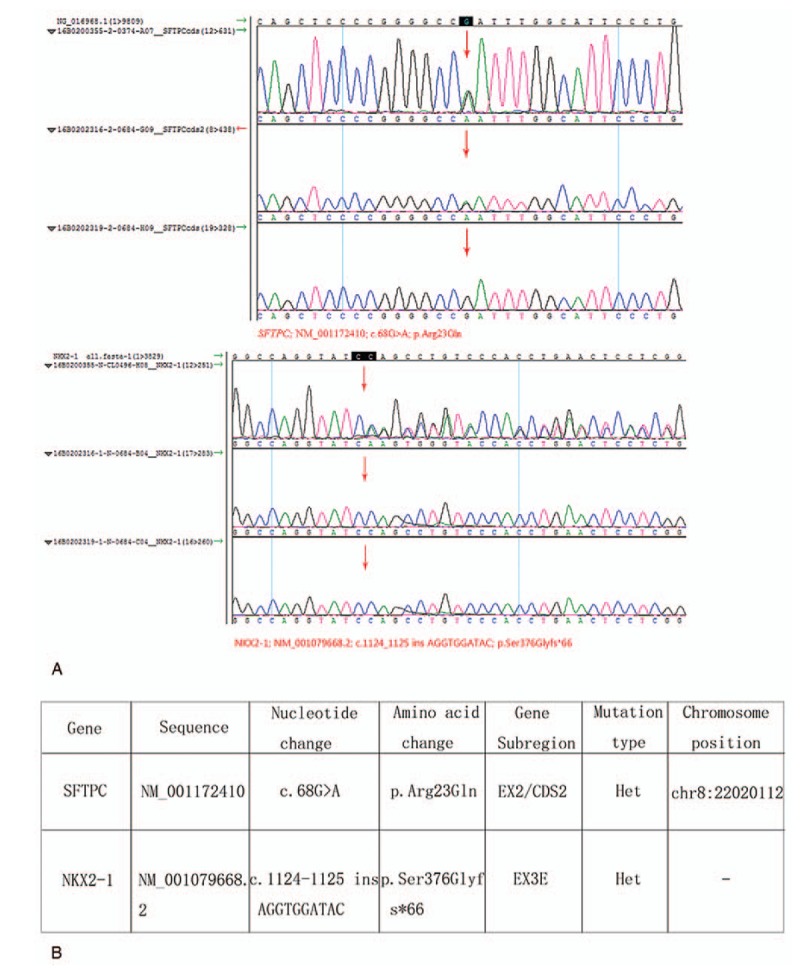
A, Chromatograms obtained from the genetic analysis of the patient (16B0200355) and his parents (father 16B0202316 and mother 16B0202319). The corresponding chromatograms of exon 3E of NKX2-1 showing c.1124_1125insAGGTGGATAC mutation (arrow). The upper panels represent the rare c.68G > C mutation within CDS2 of SFTPC. B, Form illustrated gene change comparisons between SFTPC and NKX2-1 mutation. SFTPC = surfactant protein C gene.

Sequencing of the NKX2–1 gene led to the detection of a novel heterozygous 1-base pair insertion (c.1124_1125insAGGTGGATAC) in exon 3E resulting in a frame shift starting at serine 376(p.Ser376Glyfs∗66). The protein encoded by the NKX2–1 gene loses its stop codon and causes the translation to extend to position 440. A rare c.68G > C mutation was identified in this patient within the CDS2 segment of the SFTPC gene. The missense mutation resulted in substitution of an arginine residue by glutamate at codon 23 (p.Arg23Gln). The mutation was also detected in his father, as shown in Fig. 2A. The gene change comparisons between the SFTPC and NKX2-1 mutations are shown in Fig. 2B.

## 4 Discussion

We reported a fatal outcome in an infant with ILD, persistent low oxyhemoglobin saturation, congenital hypothyroidism, feeding difficulty, irritability, and convulsion, which constitute NKX2-1-related disorders. Although several heterozygous mutations have been reported, this is the first study to report mutations of both, NKX2-1 and SFTPC genes, which ultimately led to the patient's death.

This patient demonstrated lung, thyroid, and neurological disorders, similar to several reports on NKX2-1-related disorders. At birth, the baby had pneumonitis and pneumothorax, which further developed into interstitial pneumonia with low oxyhemoglobin saturation in just 1 month. At the same time, subclinical hypothyroidism was found on neonatal screening. The serum TSH levels normalized after 8 days of levothyroxine treatment. The infant gradually developed neurological symptoms including feeding difficulty and irritability, which were difficult to treat despite hospitalization. After 2 months’ hospitalization, the baby died from recurrent pulmonary infections and fatal low oxyhemoglobin saturation. It is widely known that some patients succumb to neonatal respiratory distress syndrome in the early stage, some patients subsequently develop interstitial lung disease in childhood, while pulmonary disease is mild or absent in others. The variety in clinical manifestation may be attributed to tissue specificity, environmental factors, or coexistence of gene mutations.^[8]^ However, in this case, we found that the baby died from severe interstitial pneumonia, which was probably caused by the superimposition effect of 2 gene mutations, which shortened the disease course.

NKX2-1 influences lung morphology, pulmonary epithelial cells and their function, especially in regulating the diversity of surfactant protein (SP) genes.^[9]^ Surfactants can lower surface tension at the air–liquid interface, so that the alveoli do not collapse at end-expiration. The function of SP-C is unclear, but it is thought to be closely related to the functional integrity of the lungs.^[10]^ Surfactant stabilization rather than synthesis is its main function.^[11]^ SP-C can also defend against lipopolysaccharide-induced pulmonary inflammation.^[12]^ SFTPC mutations have been found to induce ILD. SP-C gene heterozygous mutations produce aberrant proSP-C proteins. The aberrant proSP-C proteins weaken normal proSP-C proteins production, which have some functional activity. The aberrant protein is difficult to degrade and toxic to type II alveolar epithelial cells that induce the inflammatory response. Other factors including viral infection, hypoxia, and drugs may induce aberrations in proSP-C proteins.^[12,13]^ SP-C mutations result in leukocytic dysfunction, which include loss of toll-like receptor 3 (TLR3) modulation, and IL-8 secretion. It also affects chemokine (C-C motif) receptor 2 (CCR2) and chemokine (C-X-C motif) receptor (CXCR1), which cause uncontrolled inflammation and fibroblast proliferation.^[6]^ This is the first report to demonstrate genic mutations of both NKX2.1 and SFTPC, which were ultimately fatal. Thyroid transcription factor-1 reportedly regulates SFTPC transcription directly or by connecting the transcriptional co-activator with the PDZ-binding motif.^[14]^ In fact, the combination of 2 mutations can damage pulmonary SP homeostasis and gas exchange, lower oxyhemoglobin saturation, and cause recurrent pulmonary infection and rapid progression of lung disease.

We found a new mutation of NKX2-1 (c.1124_1125insAGGTGGATAC) and a rare mutation of SFTPC (c.68G > C) in our patient. This mutation of NKX2-1 has not been reported in the literature and has not been detected in the genome sequencing database of the normal population. The probability of occurrence of this mutated locus of SFTPC in the normal population is very low and has no clear clinical significance. The mutation of NKX2-1 was found only in the patient, but the SFTPC mutation could have been inherited from his father. However, his father had no relevant clinical manifestations. Hence, the NKX2-1 mutation may have affected SP-C function and caused recurrent pulmonary infection or other lung diseases. A disease caused by 2 mutations is extremely rare and has seldom been reported. This is the first report of brain-lung-thyroid syndrome concomitant with SFTPC mutation, which caused severe ILD and persistent low oxyhemoglobin saturation, which caused the patient's death.

In conclusion, previously published reports have shown that NKX2-1-mutation was associated with phenotypic variation and a spectrum of clinical manifestations including benign hereditary chorea, hypothyroidism, chronic ILD, and respiratory distress syndrome. Herein, we have reported a combined mutation of NKX2-1 and SFTPC, which caused recurrent pulmonary infection and fatal low oxyhemoglobin saturation, and ultimately led to the patient's death. Besides the novel mutation of NKX2-1, the patient also presented with mild hypothyroidism and neurological symptoms. This is the first study to report a disease caused by mutation of both NKX2-1 and SFTPC genes. Therefore, this disease should be considered when idiopathic interstitial pneumonia is encountered.

## Author contributions

**Conceptualization:** Zhou Jiang, Guangyong Ye.

**Data curation:** Rui Gu.

**Funding acquisition:** Zhou Jiang, Yimin Zhou.

**Writing – original draft:** Rui Gu.

**Writing – review & editing:** Zhou Jiang.
